# The Time Window for Therapy with Peptide Nanofibers Combined with Autologous Bone Marrow Cells in Pigs after Acute Myocardial Infarction

**DOI:** 10.1371/journal.pone.0115430

**Published:** 2015-03-10

**Authors:** Ming-Yao Chang, Chih-Han Chang, Chien-Hsi Chen, Bill Cheng, Yi-Dong Lin, Chwan-Yau Luo, Hua-Lin Wu, Yu-Jen Yang, Jyh-Hong Chen, Patrick C. H. Hsieh

**Affiliations:** 1 Department of Biomedical Engineering, National Cheng Kung University, Tainan, Taiwan; 2 Institute of Biomedical Sciences, Academia Sinica, Taipei, Taiwan; 3 Department of Surgery, National Cheng Kung University & Hospital, Tainan, Taiwan; 4 Department of Biochemistry and Molecular Biology, National Cheng Kung University, Tainan, Taiwan; 5 Department of Internal Medicine, National Cheng Kung University & Hospital, Tainan, Taiwan; University of Cincinnati, College of Medicine, UNITED STATES

## Abstract

**Background:**

We previously showed that injection of peptide nanofibers (NF) combined with autologous bone marrow mononuclear cells (MNC) immediately after coronary artery ligation improves cardiac performance in pigs. To evaluate the clinical feasibility, this study was performed to determine the therapeutic time window for NF/MNC therapy in acute myocardial infarction (MI).

**Methods and Results:**

A total of 45 adult minipigs were randomly grouped into 7 groups: sham or MI plus treatment with NS (normal saline), or NF or MNC alone at 1 day (1D) post-MI, or NF/MNC at 1, 4, or 7 days post-MI (N≥6). Cardiac function was assessed by echocardiography and ventricular catheterization. Compared with the NS control, pigs treated with NF/MNC at 1 day post-MI (NF/MC-1D) had the greatest improvement in left ventricle ejection fraction (LVEF; 55.1±1.6%; P<0.01 vs. NS) 2 months after MI. In contrast, pigs treated with either NF/MNC-4D or NF/MNC-7D showed 48.9±0.8% (P<0.05 vs. NS) and 43.5±2.3% (n.s. vs. NS) improvements, respectively. The +dP/dt and -dP/dt, infarct size and interstitial collagen content were also improved in the NF/MNC-1D and -4D groups but not in the -7D group. Mechanistically, MNC quality and the states of systemic inflammation and damaged heart tissue influence the therapeutic efficiency of NF/MNC therapy, as revealed by another independent study using 16 pigs.

**Conclusions:**

Injection of NF/MNC at 1 or 4 days, but not at 7 days post-MI, improves cardiac performance and prevents ventricular remodeling, confirming the importance of early intervention when using this therapy for acute MI.

## Introduction

The limited regenerative ability of mammalian cardiomyocytes renders them vulnerable to myocardial infarction (MI), which results in irreversible cardiomyocyte death. This leads to cardiac dysfunction and, ultimately, heart failure. Recently, stem cell therapy has shown promising outcomes and has opened a new window for cardiac reparative and regenerative medicine [1]. Among the cell types used for cardiac cell therapy, the bone marrow cell (BMC) is of particular interest because of the availability of abundant autologous BMCs for transplantation [2–4]. However, although BMC therapy can achieve a certain level of cardiac protection, there are still many obstacles to the successful treatment of MI by this approach. For example, the loss of intramyocardially injected cells is inevitable because the heart is a constantly contracting organ. Previous studies have demonstrated that only 1.3~2.6% of the administered cells were detected 1.5 hours after the injection [5]. It is now widely accepted that the effectiveness of cell therapy in improving cardiac function heavily depends on the cell retention numbers [6].

Another important issue is the timing of cell delivery. Specifically, even though BMC transplantation has been shown to be safe [7], the effective therapeutic time window of this treatment has rarely been explored [8,9]. Although some clinical data show improved left ventricle ejection fraction (LVEF), scar size, and ventricle volume in patients injected with BMCs within 7 days of MI [10], other studies indicate that the delivery of bone marrow mononuclear cells (MNCs) in early or later time points after acute MI has no significant benefits on cardiac function [11–13].

We have used a hydrogel-based tissue engineering approach to promote cell retention and recruitment for post-MI cardiac repair in rats and pigs [14–16]. In a recent pig study, we reported that injecting self-assembling peptide nanofibers (NF) combined with autologous MNC increases cell retention/survival by approximately 8-fold at 1 month post-MI, improving cardiac performance and preventing remodeling [17]. To further investigate the therapeutic time window of this treatment in large animals, here we tested whether our delayed NF/MNC therapy improves cardiac function in pigs with acute MI.

## Methods

### Experimental Animals

All animal research procedures were approved by the National Cheng Kung University Institutional Animal Care and Use Committee, in which the Committee reviews all animal works based on the guidelines established by US National Institutes of Health and Federation of European Laboratory Animal Science Association. Sexually mature Lanyu minipigs (~5 months old, mean body weight 23.88 kg) from National TaiTung Animal Propagation Station were used. Anesthesia was administered to all animals before any surgeries and *in vivo* measurements. After an overnight fast, the pigs were injected with Zoletil (12.5 mg/kg; Virbac, France), Rompun (0.2 ml/kg; Bayer Healthcare, Germany) and atropine (0.05 mg/kg; TBC, Taiwan) before intubation. They were attached to a respirator for intermittent positive pressure ventilation with a mixture of oxygen, air and Isoflurane (1.5 to 2%; Baxter Healthcare, Guayama, PR). An indwelling needle was placed in an ear vein for continuous administration of saline and anesthetic drugs if necessary. Administration of dextrose (glucose) and electrolyte solutions was performed during all surgical procedures. After surgery, analgesics (Keto; YSP, Taiwan) and antibiotics (Ampolin; YSP, Taiwan) were administered to relieve pain and prevent infection.

### Myocardial Infarction and treatment

We first used 16 pigs to determine the damage of myocardial tissue and bone marrow cell composition at 1, 4, and 7 days post-MI. Afterward, a total of 45 animals were divided into 7 groups, and a sham operation was performed by opening the chest without coronary artery ligation. The treatment groups were defined as follows: MI+NS (normal saline), MI+NF-1D (1 day post-MI with NF), MI+MNC-1D (1 day post-MI with MNC), MI+NF/MNC-1D (1 day post-MI with NF and MNC), MI+NF/MNC-4D (4 days post-MI with NF and MNC), and MI+NF/MNC-7D (7 days post-MI with NF and MNC). MI was induced by permanent mid-left anterior descending (LAD) coronary artery ligation. At 1, 4, or 7 days post-MI, a 2 ml injection of NS or 1% NF solution with or without MNC was administered. The cell isolation and NF preparation steps were as previously described [17].

### Bone marrow cell isolation after myocardial infarction

At 1, 4, and 7 days post-MI, a bone marrow aspiration needle (Jamshidi) was used to collect 5 ml of bone marrow from the tibial bone. The bone marrow MNC isolation process was as previously described [17]. To trace these cells, MNCs were labeled with DiI (Cell tracker; Molecular Probes, Invitrogen) according to the manufacturer’s protocol. The bone marrow cell composition was evaluated by flow cytometry, using anti-CD14 (Biolegend) and anti-CD16 (Antigenix America) antibodies.

### Echocardiography and hemodynamics

**Fig 1:**
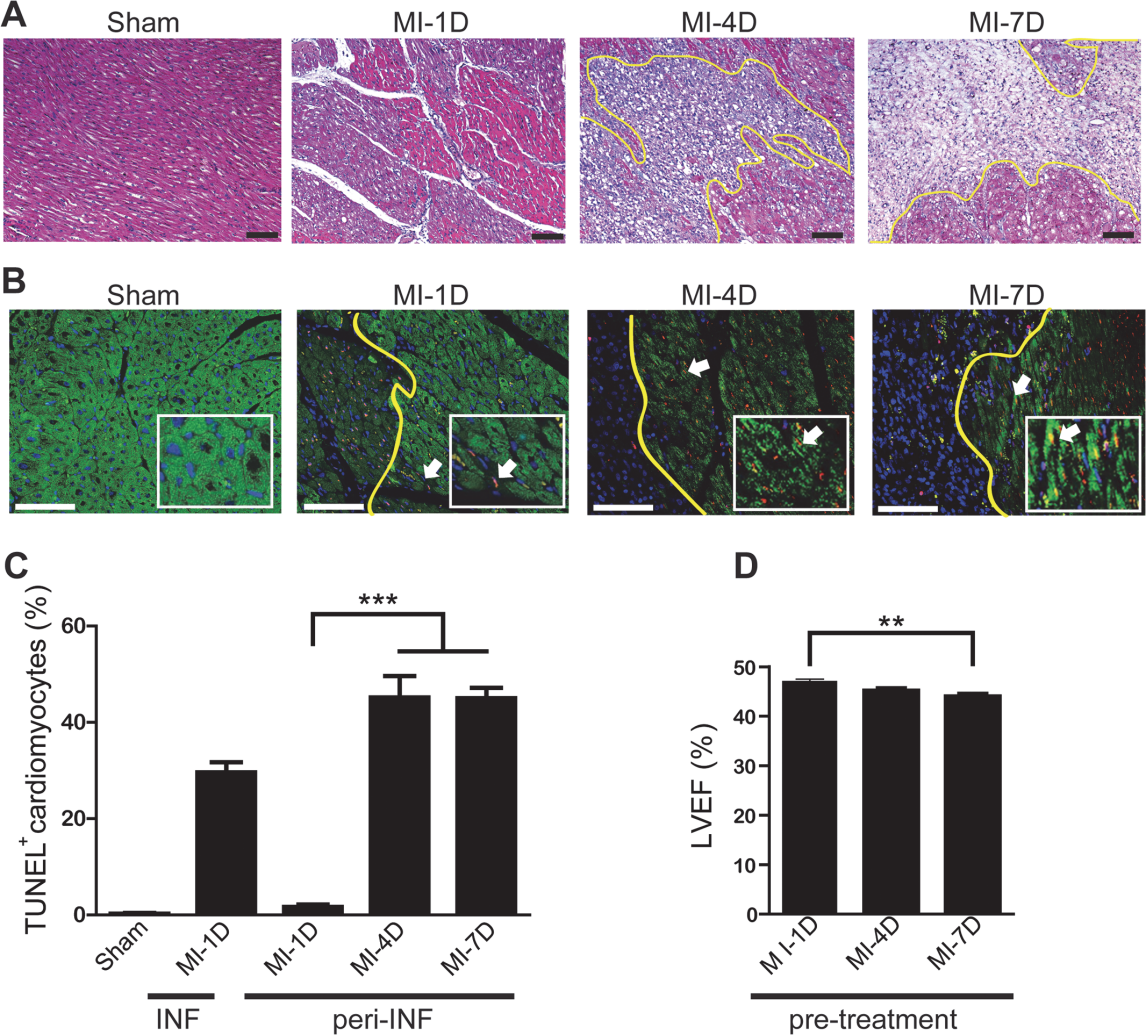
Cardiac morphology and function worsens at later time points post-infarction. **(A)** Images represent cardiomyocyte disruption and inflammatory cell infiltration in the border zone by H&E staining (10X, scale bar = 100 μm). **(B)** Images represent the infarct area (left of the yellow line) and peri-infarct border zone (right of the yellow line) at 1, 4, and 7 days after myocardial infarction (MI), as well as TUNEL staining of cardiomyocytes. Green, troponin I; red, TUNEL positive cell; blue, DAPI (20X, scale bar = 100 μm). **(C)** Quantification of cardiomyocyte apoptosis following MI by TUNEL staining. **(D)** At 1, 4, and 7 days post-infarction, the left ventricle ejection fraction (LVEF, %) was measured using echocardiography and compared between different time points with N≥5 per time point. Data are presented as the mean±SEM. **P<0.01.

The cardiac function was assessed by echocardiography before and immediately after MI (Fig. 1A), before treatment administration and 1 and 2 months after surgery using Vivid 7 with a 3.5 MHz probe (GE Healthcare, Norway). The animals were placed in a lateral decubitus position. The anesthesia condition used for echocardiography was the same as that during surgery. Parasternal long-axis views were obtained with both M-mode and 2D echo images. After 2 months, the hemodynamics was assessed by ventricular catheterization using 5.0 Fr. pressure-volume sensing catheters (Millar Instruments, Houston, TX). The left ventricle functional measurement and hemodynamic data calibration were described previously [17].

### Ratio of scar tissue and collagen content

After all *in vivo* measurements, animals were sacrificed. Hearts were harvested and the atria were removed. The heart was then cut into 5 parts from the apex to the mid-ventricle and placed upright. Images were then collected and the scar tissue (pale) was quantified by manual tracing and software calculation (Image J, NIH). The histology of the infarcted area was examined by H&E staining. Collagen content was determined using Masson’s trichrome staining. Images were taken at 5 randomly selected areas from each section, by digital microscopy at 100x magnification. The collagen content was then quantified (Axio Vision, Zeiss) with each section and averaged.

### Immunohistochemistry and fluorescence microscopy

Fixed myocardial tissue sections were deparaffinized, rehydrated and boiled in 10 mM sodium citrate (pH 6.0) for 10 minutes. This was followed by incubation with antibodies against isolectin (Molecular Probes, Invitrogen), von Willebrand factor (vWF; Proteintech Group), SM22-α (Abcam), and cardiac troponin I (DSHB) at 4°C overnight followed by incubation with FITC- or Alexa Fluor-conjugated secondary antibodies (Molecular Probes, Invitrogen). Finally, sections were incubated with DAPI (Sigma-Aldrich), and all sections were observed under a fluorescence microscope. The capillary and arteriole densities at the border zone were randomly measured (200x and 100x magnification, respectively), and blinded quantification was performed by manually counting each section. The DiI-positive cells were examined in the border and infarct zones, and the images were collected from 10 views (200x magnification). MNC differentiation into endothelial cells was quantified by manually counting DiI-positive cells that demonstrated co-staining with the vWF marker within each section. The confocal images were captured in two and three dimensions and then analyzed using NIS software (Nikon).

### TUNEL assay

For the *in vivo* TUNEL assay, the pigs were divided into four groups including sham, MI-1D, MI-4D, and MI-7D for acute MI operations. The hearts were collected and placed in 4% formaldehyde for paraffin embedded tissue sectioning. Tissue sections were stained by a TUNEL Apoptosis Detection Kit (Millipore) following manufacturer’s instructions. Sections were imaged in six randomly selected views for each heart in the border zone, and TUNEL positive cells were counted for cardiomyocyte apoptosis.

### Statistical analysis

All data were presented as mean±SEM. Statistical comparison was performed with 1-way or 2-way ANOVA. A probability value of *P*<0.05 was considered statistically significant.

## Results

### Cardiac morphology and function worsens at later time points post-infarction

The morphology of cardiac tissue post-MI was first evaluated by H&E staining. It was noticed that tissue disruption with inflammatory cell infiltration at the infarcted area became more obvious at 4 and 7 days post-MI than that at 1 day post-MI (Fig. 1A). Moreover, as demonstrated by TUNEL assay, cardiomyocytes at the peri-infarct region underwent apoptosis at 1 day post-MI, which remained detectable at 4 and 7 days post-MI (Fig. 1B and 1C). Prior to NF/MNC administration, the LVEF at 1, 4, and 7 days post-MI was measured using echocardiography. The results showed that the LVEF at 7 days post-MI was significantly less than the LVEF measured at 1 day post-MI (Fig. 1D). These results suggest a progressive cardiac dysfunction and pathological destruction during the first week post-infarction.

### NF/MNC protects myocardial function depending on the injection time

**Fig 2:**
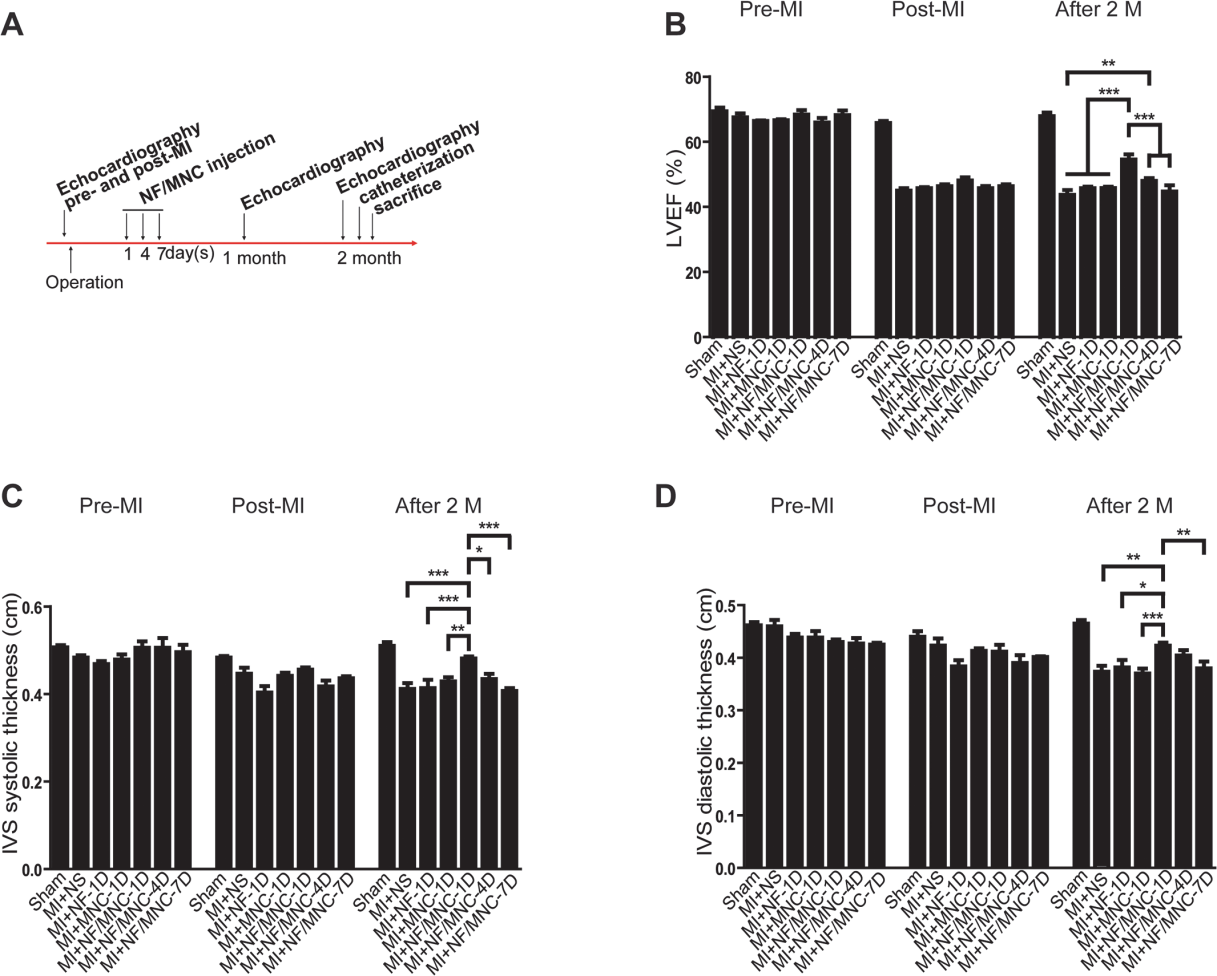
NF/MNC injection protects myocardial function depending on the injection time. **(A)** Following surgical ligation of the mid-left anterior descending coronary artery to induce MI, the pigs underwent intramyocardial injection of NF and/or MNC at 1, 4, or 7 days and were sacrificed at 2 months after MI. Echocardiography and ventricular catheterization were used to measure cardiac function. **(B)** Showing are histograms of the left ventricle ejection fraction (LVEF, %), **(C)** the interventricular septum (IVS) systolic, and **(D)** diastolic thicknesses. Data are presented as the mean±SEM. *P<0.05, **P<0.01, ***P<0.001.

The experimental flow chart is shown in Fig. 2A. Again we confirmed by echocardiography that the LVEF decreased significantly immediately after the MI surgery prior to the injection of any materials (Fig. 2B). Autologous MNCs were extracted, mixed with NFs and injected into the infarct area at 1, 4, or 7 days post-MI. Two months after MI, echocardiography revealed that the LVEF of the MI+NF/MNC-1D group (55.1±1.6%) was significantly higher than that of the NS (44.3±1.4%), NF-1D (46.5±0.5%), and MNC-1D (46.3±0.4%) groups (*P*<0.001, Fig. 2B). Interestingly, the MI+NF/MNC-4D group (48.5±0.8%) also had a greater LVEF (*P*<0.01), but the MI+NF/MNC-7D group (45.3±1.9%) demonstrated no improvement in the LVEF compared with the control group or the groups that received NF or MNC alone. Consistent with these results, the interventricular septum (IVS) systolic and diastolic thicknesses, which were verified by echocardiography, also displayed a similar trend of improvement due to NF/MNC injection at 1 day or 4 days, but not at 7 days post-MI (Fig. 2C and 2D).

**Fig 3:**
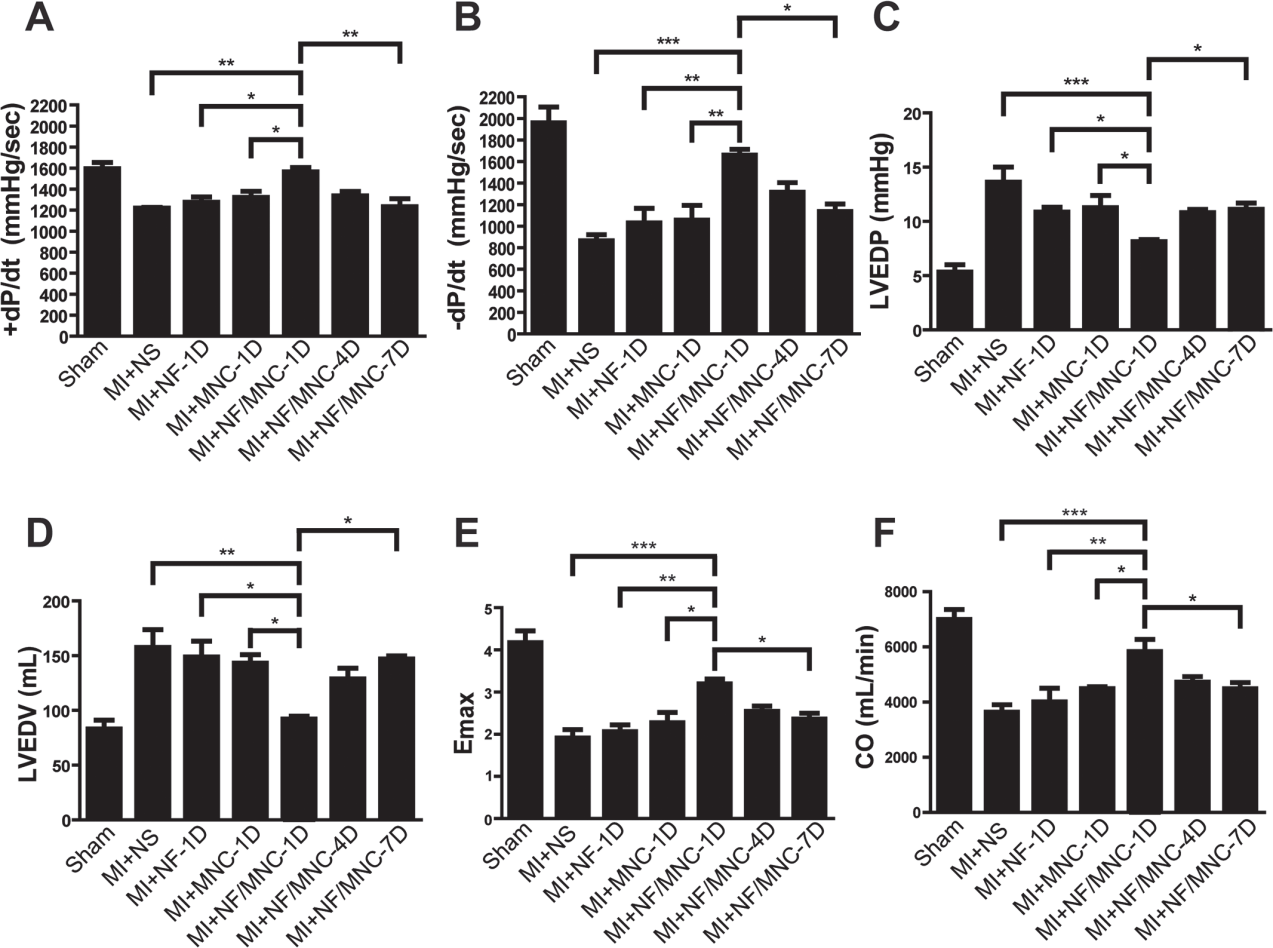
NF/MNC injection improves cardiac hemodynamics depending on the injection time. **(A)** Hemodynamic parameters in pigs at 2 months post-MI, including pressure increment (+dP/dt), **(B)** pressure decrement (-dP/dt), **(C)** left ventricle end-diastolic pressure (LVEDP), and **(D)** volume (LVEDV), **(E)** maximum chamber elasticity (Emax), and **(F)** cardiac output (CO). Data are presented as the mean±SEM. *P<0.05, **P<0.01, ***P<0.001.

In agreement with the echocardiographic results, the hemodynamic analysis also showed that the NF/MNC-1D treatment effectively preserved the systolic (+dP/dt) and diastolic (-dP/dt) functions compared with the NS, NF-1D, MNC-1D, or NF/MNC-7D treatments. However, no significant differences were observed between the NF/MNC-1D and NF/MNC-4D (Fig. 3A and 3B). Additionally, the injection of NF/MNC-1D prevented LV dilation and heart failure, as indicated by decreased LV-end diastolic pressure (LVEDP, Fig. 3C) and volume (LVEDV, Fig. 3D) and enhanced maximum LV elasticity (Emax, Fig. 3E). All of these outcomes contributed to the improvement of LV contraction and maintained cardiac output 2 months post-MI (Fig. 3F). Furthermore, the data consistently demonstrated a pattern in which the heart function decreased when NF/MNC was injected at 7 days post-MI (Fig. 3A and 3B), and that the heart was also found to be dilated in the NF/MNC-7D group (Fig. 3C and 3D). Decreases in the contraction properties and the cardiac output were also observed in this group (Fig. 3Eand 3F).

### NF/MNC injection at early time points post-MI decreases infarct size and prevents cardiac fibrosis

**Fig 4:**
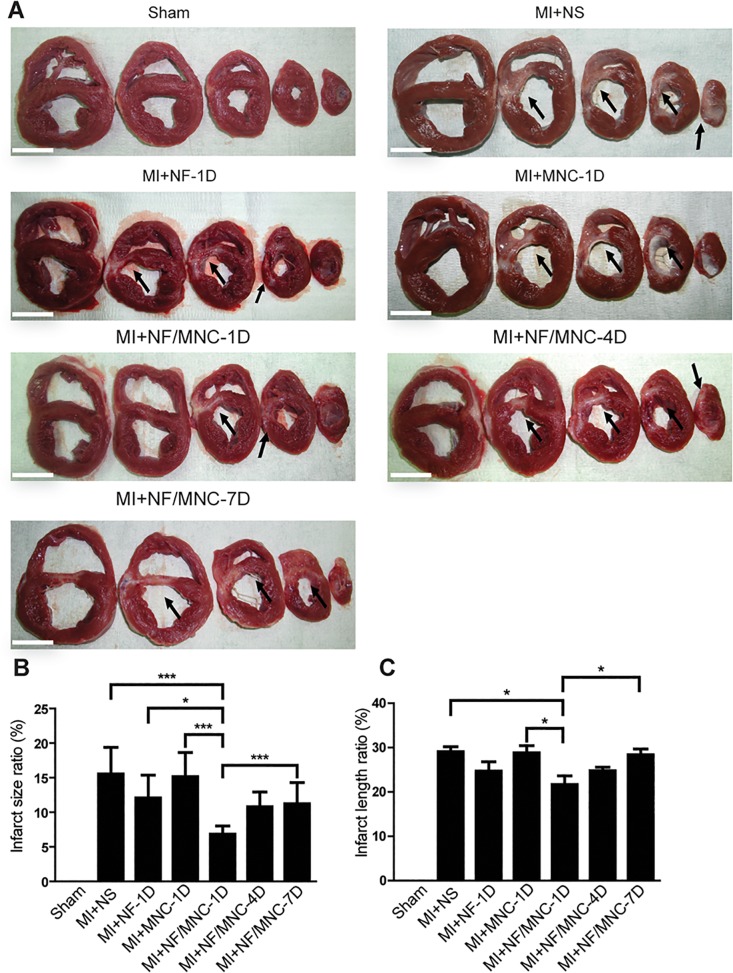
NF/MNC injection at early time points post-MI decreases infarct size. (**A)** Representative images of fresh heart sections from the apex to the mid-ventricle from each group. Arrows indicate the infarcted area (scale bar = 3 cm). **(B)** Statistical analysis of infarct size and **(C)** infarct length. Data are presented as the mean±SEM. *P<0.05, ***P<0.001.

**Fig 5:**
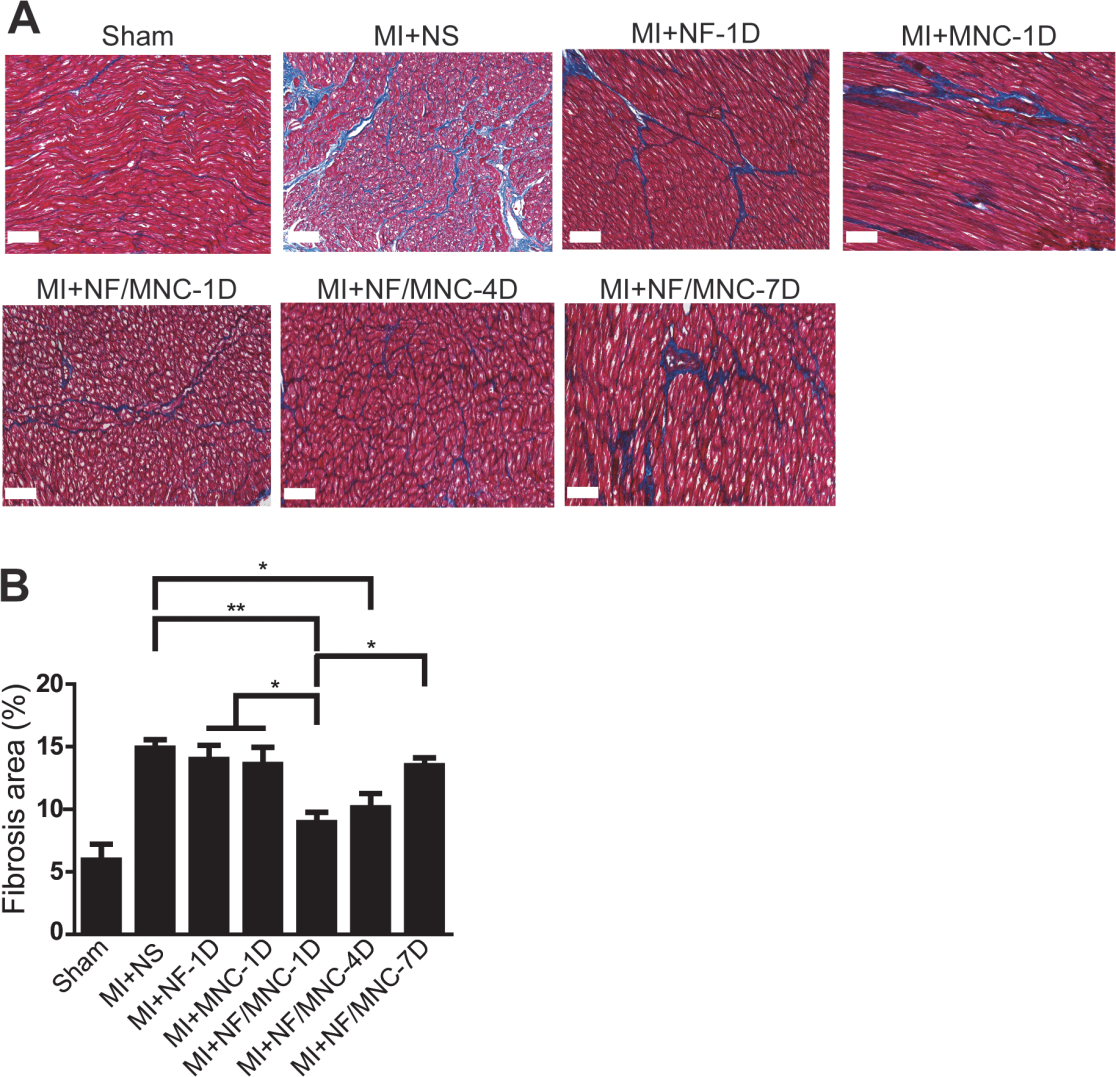
NF/MNC injection at early time points post-MI prevents cardiac fibrosis. (**A)** Representative images of the collagen content at the non-infarct area from each group. **(B)** Statistical analysis of the collagen content. Data are presented as the mean±SEM. *P<0.05, **P<0.01, ***P<0.001. Scale bar = 100 μm.

We collected heart tissues from each group 2 months post-MI and sectioned the hearts into 5 portions from the apex to mid-LV (Fig. 4A).We found that the infarct size in the MI+NF/MNC-1D group was smaller than the infarct sizes in the MI+NS, MI+NF-1D, MI+MNC-1D, and MI+NF/MNC-7D groups (Fig. 4B). We also measured the infarct length ratio (%) and obtained similar patterns to the changes in infarct size over time. The infarct length ratio of the 1 day post-MI group was significantly smaller than the 4 or 7 days group (Fig. 4C). While the difference is not significant, infarct length ratio from the 4 days group is smaller than the 7 days group (Fig. 4C). These results display a similar pattern with respect to cardiac functions. We further assessed the interstitial fibrosis at the remote area using trichrome staining (Fig. 5A). Not surprisingly, the injection of NF/MNC at 1 or 4 days post-MI resulted in significantly less collagen deposition. In contrast, collagen level increased significantly after the NF/MNC treatment at 7 days post-MI (Fig. 5B). Likewise, the fibrosis formation at the border area decreased significantly in the MI+NF/MNC-1D and-4D groups, but not in MI+NF/MNC-7D group (S1 Fig.). Together, these results indicate a strong correlation between a delayed NF/MNC treatment time and infarct size as well as cardiac fibrosis after MI.

### NF/MNC injection increases transplant cell retention after MI

**Fig 6:**
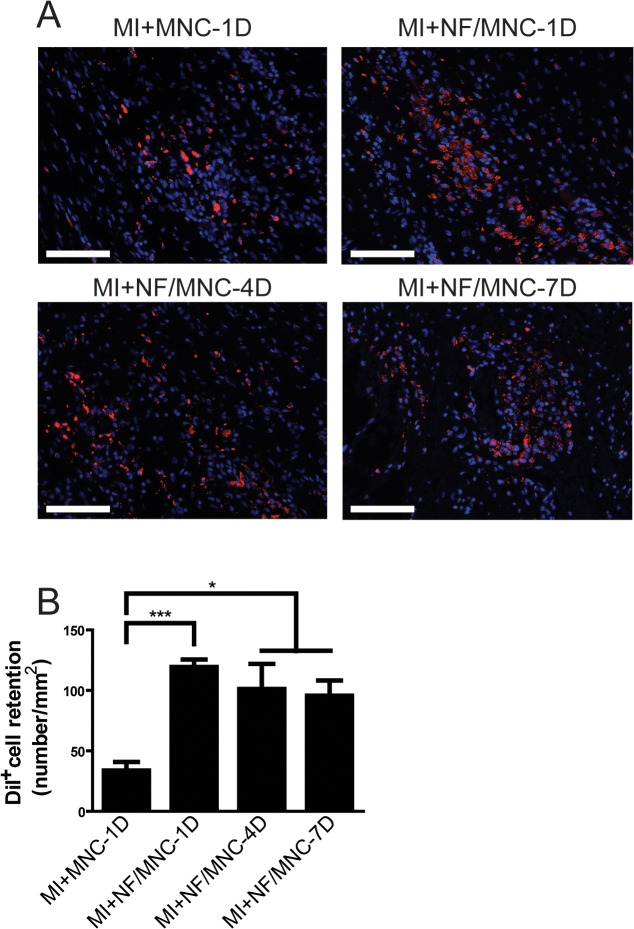
NF/MNC injection increases transplant cell retention after MI. **(A)** Representative DiI^+^ MNC (red) after injection with or without NF at 1, 4, and 7 days post-infarction. Nuclei were stained using DAPI. **(B)** The statistics of DiI^+^ MNC retention ratio. Data are presented as the mean±SEM. *P<0.05, ***P<0.001. Scale bar = 100 μm.

Cell retention is one of the major determining factors for effective and successful cell transplantation. Therefore, we compared the amount of cells retained 2 months post-MI for each of the injection groups (Fig. 6A). We found that the injection of NF/MNC significantly increased MNC retention even at 2 months post-MI compared with the injection of MNC alone (Fig. 6B). Interestingly, although the retention ratio exhibited a decreasing trend with time, there was no significant difference among the different injection time points (Fig. 6B). These results were further confirmed by confocal microscopy showing co-localization of the Dil and DAPI staining (S2 Fig.).

### Early stage NF/MNC injection increases capillary and arteriole densities at the border zone

**Fig 7:**
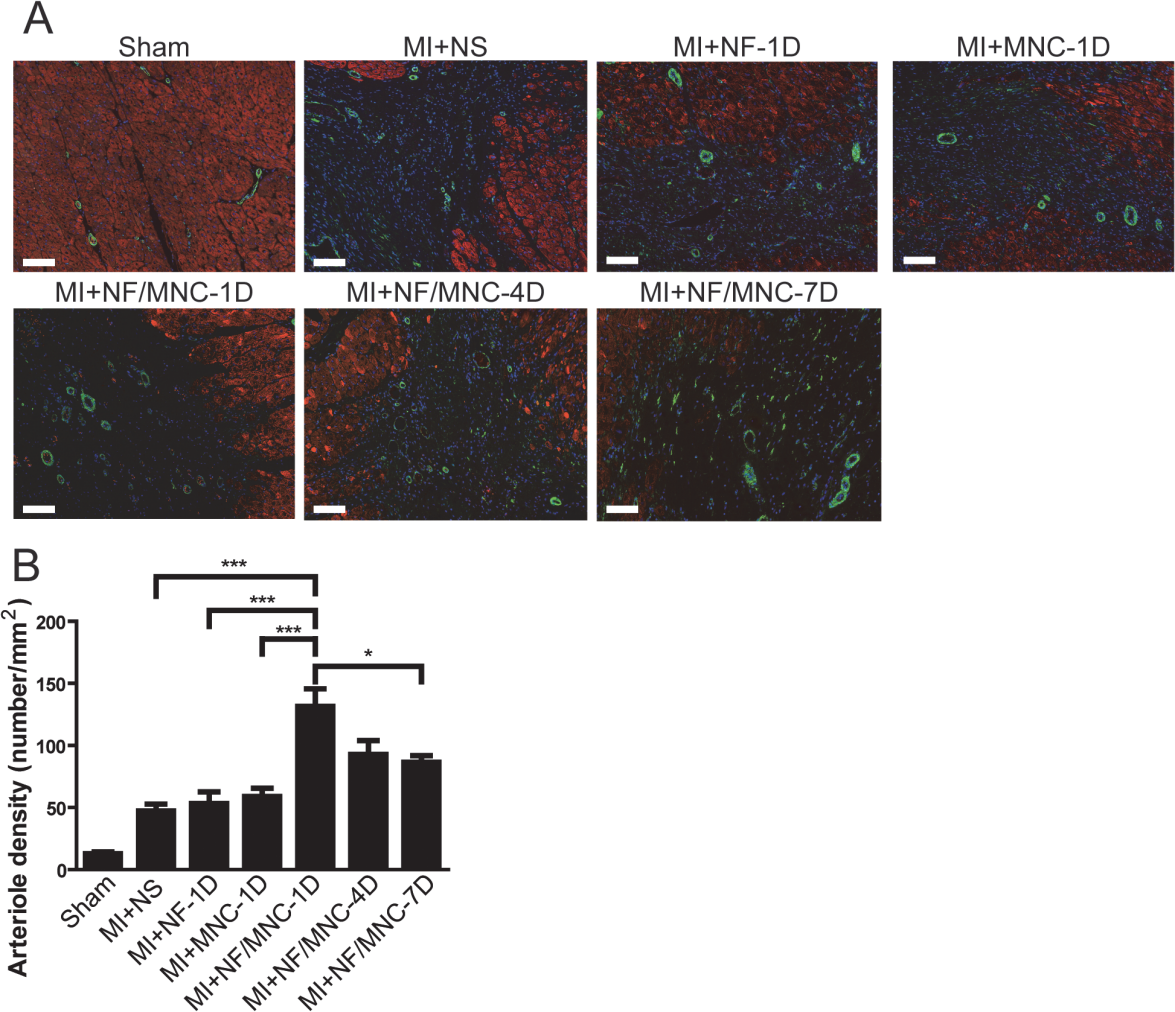
Early stage NF/MNC injection increases capillary density at the border zone. **(A)** Representative immunostaining of isolectin (green) overlapped with troponin I (red) in the peri-infarct border zone. Nuclei were stained using DAPI (blue). **(B)** Quantification of the capillary density at the border zone. Data are presented as the mean±SEM. *P<0.05, **P<0.01, ***P<0.001. Scale bar = 100 μm.

To elucidate the underlying mechanism that ameliorated cardiac function after infarction, we utilized immunofluorescence staining of isolectin (Fig. 7A) and smooth muscle 22-α (SM22α, S3 Fig.) to quantify the border zone capillary and arteriole densities, respectively. Both the capillary and arteriole densities were significantly greater in the MI+NF/MNC-1D group than in the MI+NS, MI+NF-1D, and MI+MNC-1D groups, which verifies the observed cardiac function improvement after 2 months. Surprisingly, the capillary and arteriole densities decreased at the later injection time points as opposed to the MI+NF/MNC-1D group, which had the greatest capillary (Fig. 7B) and arteriole densities (S3B Fig.). This finding is also in agreement with the better cardiac outcome seen at 2 months with the NF/MNC-1D treatment than with the NF/MNC-4D or NF/MNC-7D treatments.

### Acute myocardial infarction changes bone marrow cell composition and decreases endothelial differentiation capability

**Fig 8:**
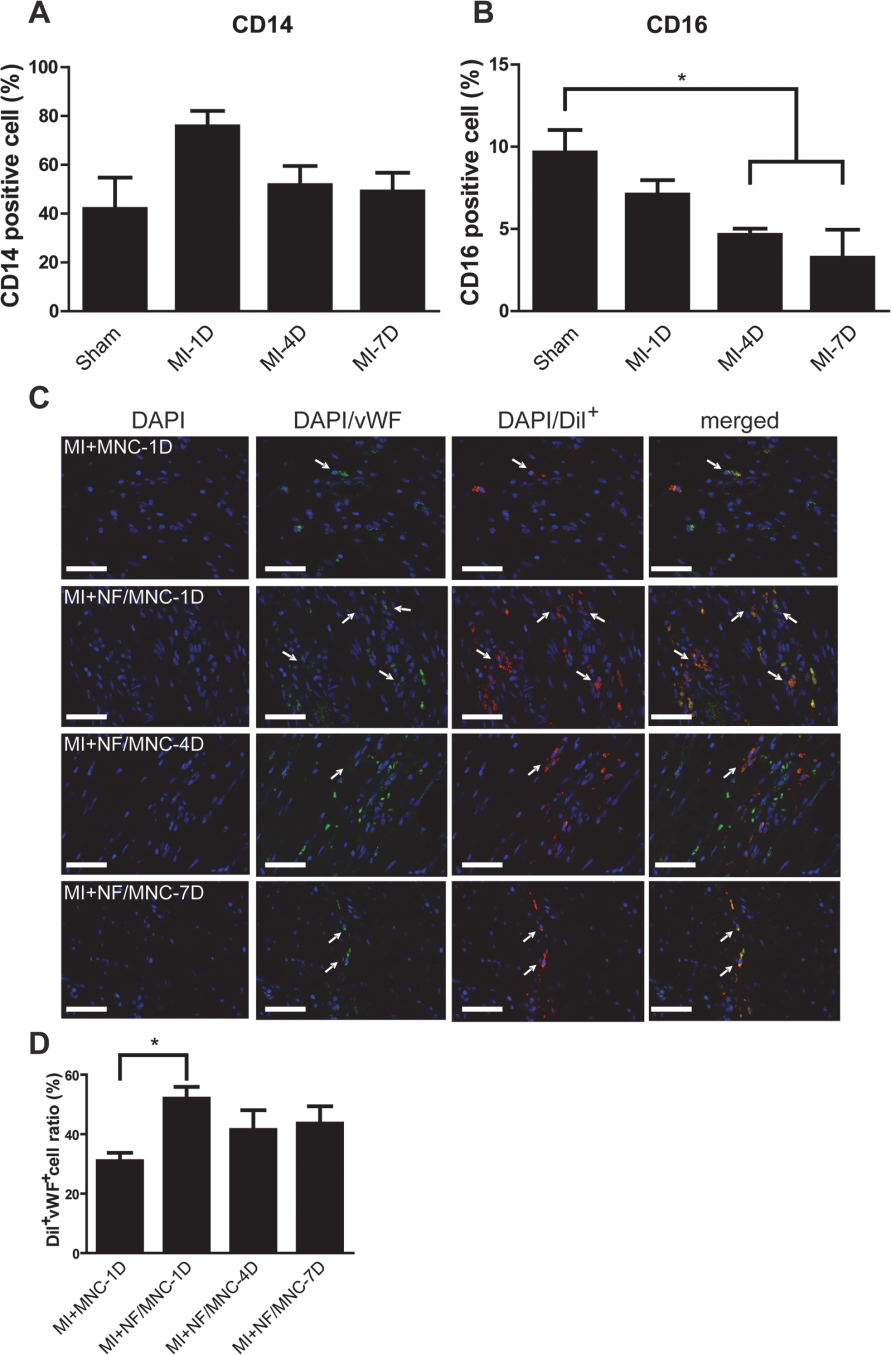
Acute myocardial infarction changes bone marrow cell composition and decreases endothelial differentiation capability. **(A)** CD14 positive cells increased at day 1 post-MI while the CD16 positive cell decreased at day 4 and day 7 after infarction in **B** with n ≥3 per groups. *P <0.05. **(C)** Representative images of DiI fluorescence (red) overlapped with immunostaining for von Willebrand factor (vWF, green) from different groups. Nuclei were stained using DAPI (blue). **(D)** Quantification of the ratio of cells stained positively with vWF. Data are presented as the mean±SEM. *P<0.05. Scale bar = 50 μm.

Since the MNCs administered to the MI porcine models were extracted from the same animals after MI, the effect of MI on the bone marrow cell composition was analyzed. Bone marrow samples were taken at 1, 4, and 7 days post-MI. The number of cells that stained positively for CD14 and CD16 were examined (Fig. 8A and 8B). The percentage of CD14^+^ cells increased at 1 day post-MI, and then decreased to the similar level as the sham group at 4 and 7 days. In contrast, CD16^+^ cells showed a decreasing trend from 1 to 7 days post-MI. The ability of MNC differentiation into vascular cells was also examined. It was noticed that the MNCs administered at 1 day post-MI showed the greatest rate of differentiation into endothelial cells compared to those injected at 4 and 7 days (Fig. 8C, D and S4 Fig.).

## Discussion

Although our previous study demonstrated that NF/MNC injection exerts beneficial effects in porcine MI models, the optimal therapeutic time window of this treatment was not determined [17]. Therefore, in the present study we designed a delayed treatment model where the therapy was administered at 1, 4, and 7 days post-MI. We demonstrated that the combination of NF with MNC improves cardiac performance when compared with the injection of NF or MNC alone. Importantly, the therapeutic effects of the injections made at 1 or 4 days post-MI persisted for at least 2 months.

Ideally, the result would be more comprehensive if MI+NF-4D and MI+NF-7D were also included in the study. However, given that a significant difference was already seen between MI+NF-1D and MI+NF/MNC-1D and that the cost of caring the animals, it was decided not to include those two groups. Although an improvement in cardiac function was seen from the NF/MNC injection at 4 days post-MI, but the benefit was still less than that provided by the NF/MNC delivery at 1 day. Mechanistically, we demonstrated that the number of apoptotic cardiomyocytes, and possibly the bone marrow cell composition after MI could affect the therapeutic outcome of the therapy.

This study demonstrated that apoptotic cardiomyocytes in the peri-infarct area can still be detected at 7 days post MI. It has been shown that the acute inflammatory response that happens after MI can continue for at least 1 week, with the peak response at 3 days after MI [18–20]. The present study showed a large number of inflammatory cells infiltrating the infarcted area at as early as 1 day post-MI. These recruited inflammatory cells are likely composed of neutrophils, monocytes, macrophages that are known to secret pro-inflammatory factors at the infarcted area, which can cause cardiomyocyte death at later time points post-MI [19,21,22]. It has been suggested that stem cell therapy may decrease cardiomyocyte apoptosis by paracrine effect [23,24]. Thus, it is suspected that the NF/MNC injection rescued the cardiomyocytes at the peri-infarct zone possibly by the same paracrine effect. However, NF/MNC injection on day 7 post-MI exerted less cardiac protection than the earlier injections. Therefore, as the injection time is being delayed, the number of cardiomyocyte death would reach a level where the treatment would no longer be effective.

Our study revealed that the early time point delivery of MNCs without NF didn’t improve cardiac function or decrease infarct size. This is likely due to the low number of MNCs presented at the infarcted area, as it has been demonstrated that improved cardiac function after cell transplantation is related to the cell number at the infarcted area [6]. Therefore, the results in the present study demonstrated the important role that NFs play in maximizing the number of MNCs retained at the infarct, which is in agreement with our previous studies [17,25].

It has been demonstrated that the post-MI bone marrow cell composition changes after the initial immuno-inflammatory responses took place. These cells were shown to have a negative impact on the diseased heart, leading to poorer therapeutic outcome with respect to MI [26]. In the present study, it was also noticed that the systemic inflammatory response that took place right after MI, had changed the numbers of CD14^+^ and CD16^+^ cells in the bone marrow [27]. Cells with strong CD14 expression are known to promote the activity of matrix metalloproteinases, whereas cells with strong CD16 expression are known to secret proangiogenic factors [18]. Although it remains unknown whether the administered MNCs have any negative impact on the diseased heart, the decrease in CD16^+^ cells at 4 and 7 days post-MI could explain why the therapeutic outcome of NF/MNC-4D and-7D groups is poorer than that of the NF/MNC-1D group. Thus, it is clear that not only MI itself has negative impact on the overall cardiac function, it also changes the quality of MNCs extracted from the bone marrow.

Since the present study was focusing on the therapeutic effect of MNCs and NFs in cardiac regeneration, we did not investigate the effect of the treatments on the cell composition in bone marrow. Thus, it would be an interest to see whether the changes seen in the number of CD14 and CD16 positive cells after MI can be reverse after the injection of NF/MNC. A previous study has demonstrated that NFs do not induce any systemic effect on the host after the injection [16], suggesting the material itself could not alter bone marrow cell composition.

Studies have shown that bone marrow cells extracted from patients with coronary artery disease have a poorer neovascularization capacity compared with those from healthy people [28,29]. Our study demonstrated that the administered NF/MNC at 1 day, but not at 7 days, post-MI was clearly shown to increase capillary density. However, it has been noted in human clinical trials that the increase in capillary density seen after MNC delivery does not necessarily equal to complete recovery of perfusion in the infarcted myocardium [30,31]. Therefore, whether angiogenesis that follows the administered therapy helps to improve myocardial perfusion requires further study. Nevertheless, it is clear that the MNCs from the NF/MNC-4D and NF/MNC-7D groups had lower endothelial differentiation ability compared with those from the NF/MNC-1D group. It is suspected that the changes in the bone marrow cell composition after MI may have an effect on endothelial differentiation ability, which could then lead to a poorer treatment outcome.

It has been demonstrated in mice that the animal made full recovery from MNC treatment at a defined time point, 7 days [26]. Currently, there hasn’t been any report on the full recovery time from a MNC treatment in large animal models of MI. Since the data from the porcine models of MI presented in this study were collected at 2 months post-MI, more research is still required to determine the precise recovery time frame for a large animal model. However, as our study have demonstrated, the cell composition in the bone marrow, and the quality of administered MNCs will influence the therapeutic efficacy of MNC treatment in MI.

## Conclusion

In conclusion, we have demonstrated that combined treatment with NF/MNC in a large animal model of acute MI effectively improved cardiac function. We further showed that the optimal therapeutic time window for NF/MNC treatment is within 4 days of MI. This result provides an estimated time point for the application of cardiac cell therapy, which will be useful information for future clinical testing

## Supporting Information

S1 Fig NF/MNC injection at early time points post-infarction prevents fibrosis at the border area. **(A)** Representative images of the collagen content at the border zone from each group. **(B)** Statistical analysis of the collagen content. *P<0.05, **P<0.01. Scale bar = 100 μm.
(TIF)

S2 Fig DiI and DAPI signals co-localize in the same cells 2 months after cell delivery. **(A)** Confocal laser scanning microscopy showing images of DiI dye and DAPI staining in separate or **(B)** 3D- or **(C)** 2D-merged. (**D)** Representative spectral overlap of DiI and DAPI in a cell indicated by an arrow in **(C)**. Scale bar = 10 μm.
(TIF)

S3 Fig NF/MNC injection at early time points post-infarction increases arteriole density in the peri-infarct border zone. **(A)** Representative immunostaining of smooth muscle 22-α (green) and troponin I (red) at the border zone. Nuclei were stained using DAPI (blue). (**B)** Quantification of the arteriole density at the border zone. *P<0.05, ***P<0.001. Scale bar = 100 μm.
(TIF)

S4 Fig Injected bone marrow cells differentiate into endothelial cells 2 months after infarction. **(A)** Confocal laser scanning microscopy showing images of DAPI, vWF and DiI in separate or **(B)** 3D- or **(C)** 2D-merged. **(D)** Representative spectral overlap of DAPI, vWF and DiI in a cell indicated by an arrow in **(C)**. Scale bar = 10 μm.
(TIF)
