# Supplementary figures and images for: The Time Window for Therapy with Peptide Nanofibers Combined with Autologous Bone Marrow Cells in Pigs after Acute Myocardial Infarction
Source: PLoS One. 2015 Mar 10;10(3):e0115430. doi: 10.1371/journal.pone.0115430 (PMC4355625; doi:10.1371/journal.pone.0115430)

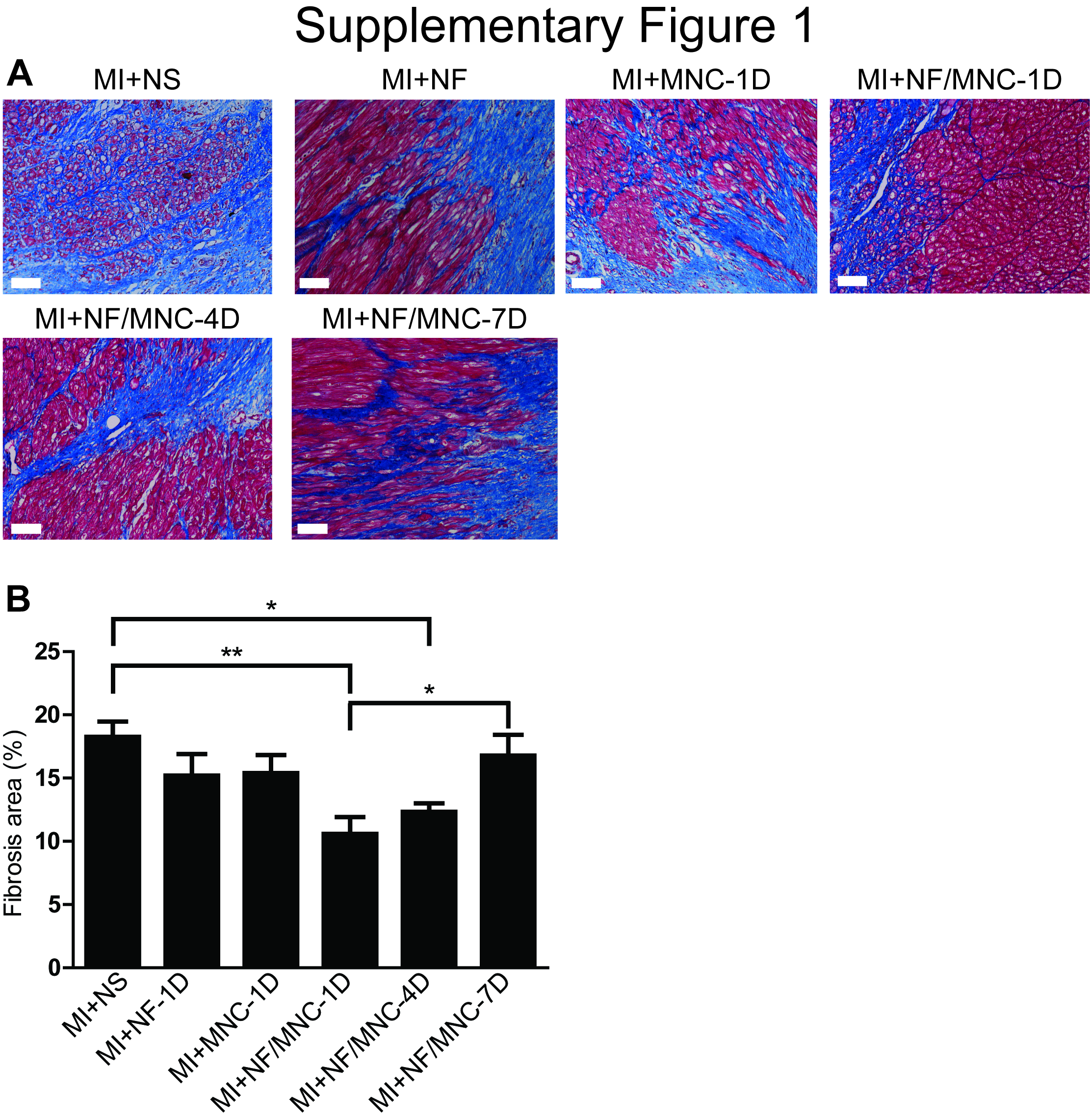

Supplement: S1 Fig — (A) Representative images of the collagen content at the border zone from each group. (B) Statistical analysis of the collagen content. *P<0.05, **P<0.01. Scale bar = 100 μm. (TIF) [file pone.0115430.s001.tif]

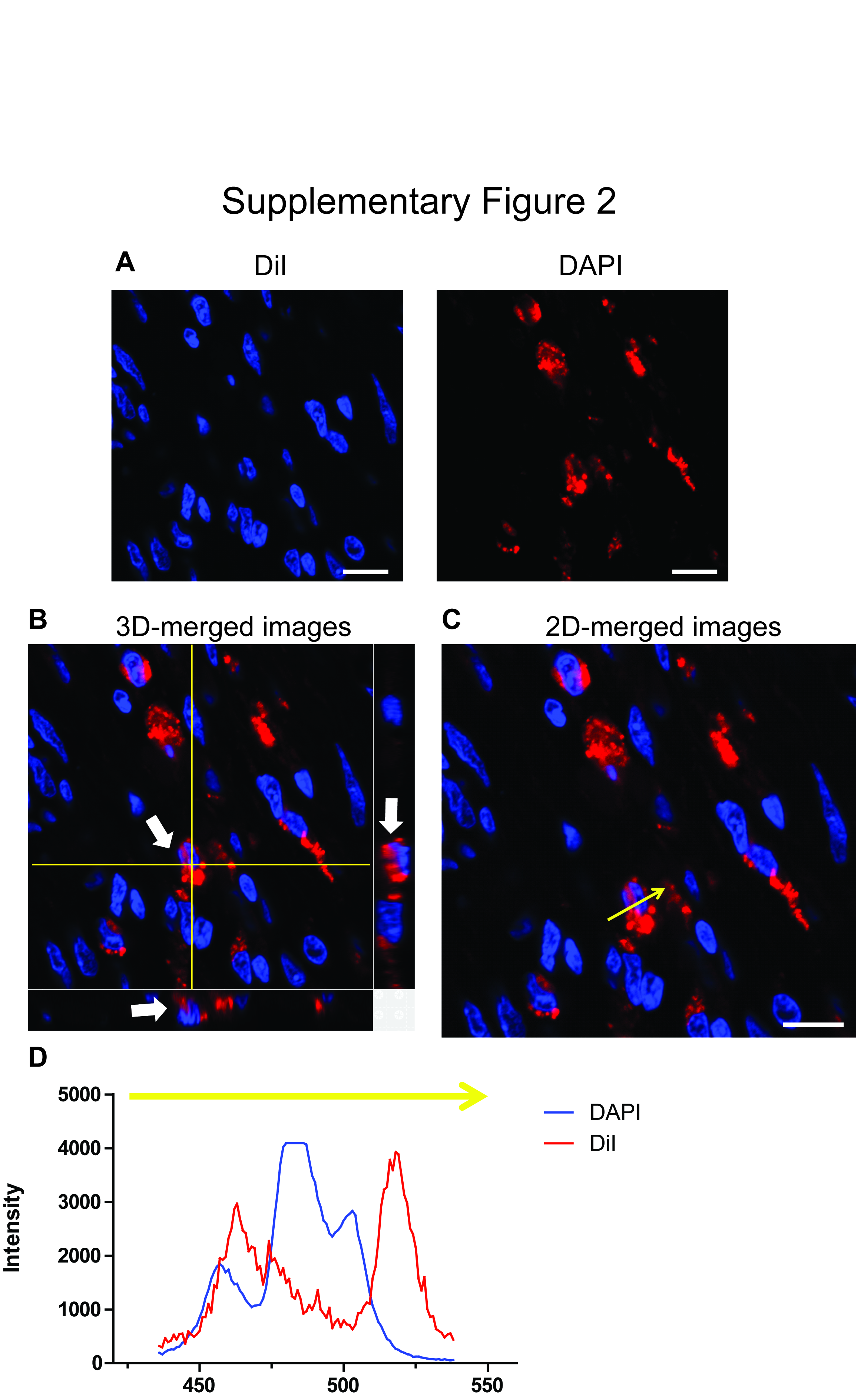

Supplement: S2 Fig — (A) Confocal laser scanning microscopy showing images of DiI dye and DAPI staining in separate or (B) 3D- or (C) 2D-merged. (D) Representative spectral overlap of DiI and DAPI in a cell indicated by an arrow in (C). Scale bar = 10 μm. (TIF) [file pone.0115430.s002.tif]

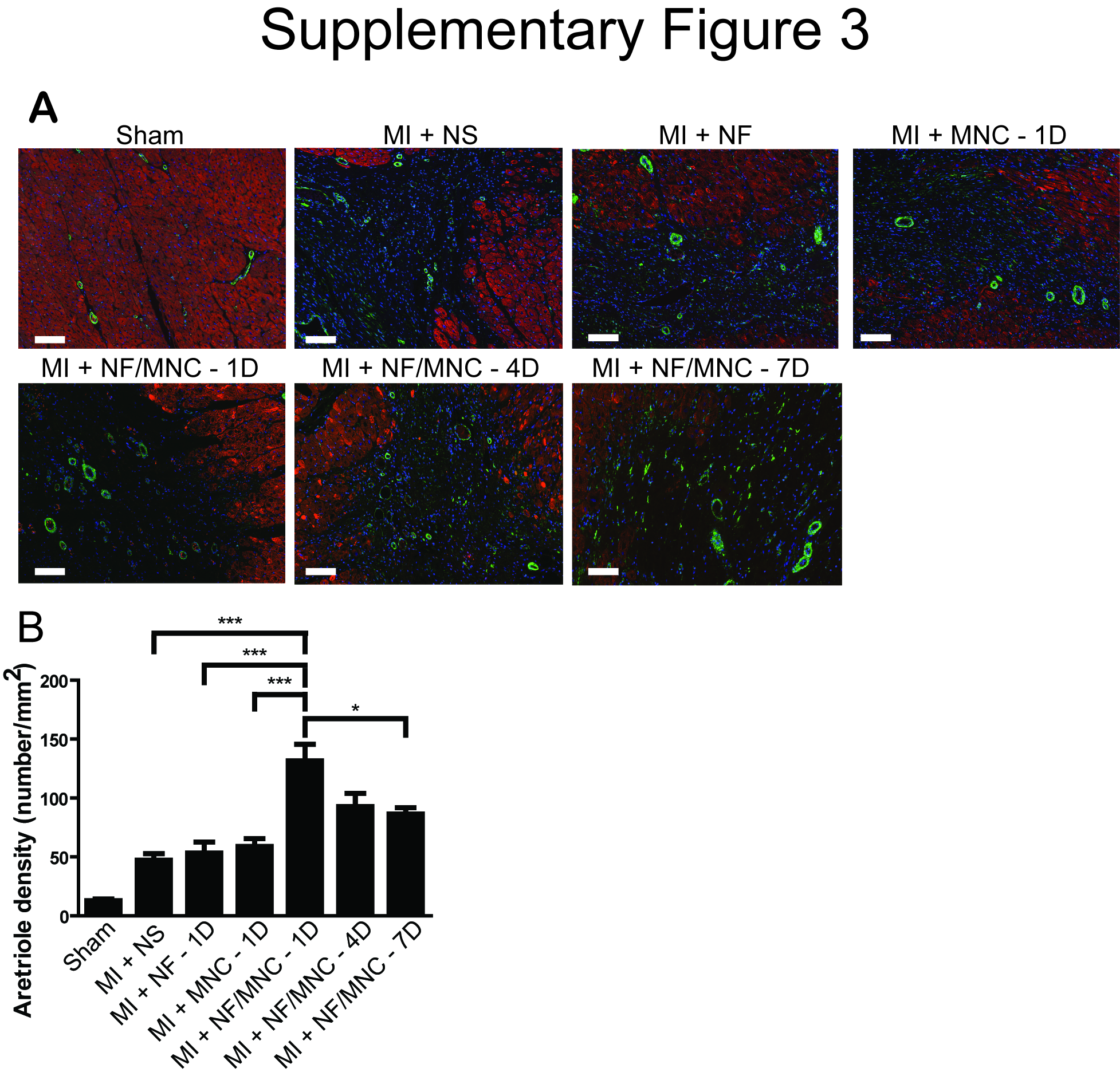

Supplement: S3 Fig — (A) Representative immunostaining of smooth muscle 22-α (green) and troponin I (red) at the border zone. Nuclei were stained using DAPI (blue). (B) Quantification of the arteriole density at the border zone. *P<0.05, ***P<0.001. Scale bar = 100 μm. (TIF) [file pone.0115430.s003.tif]

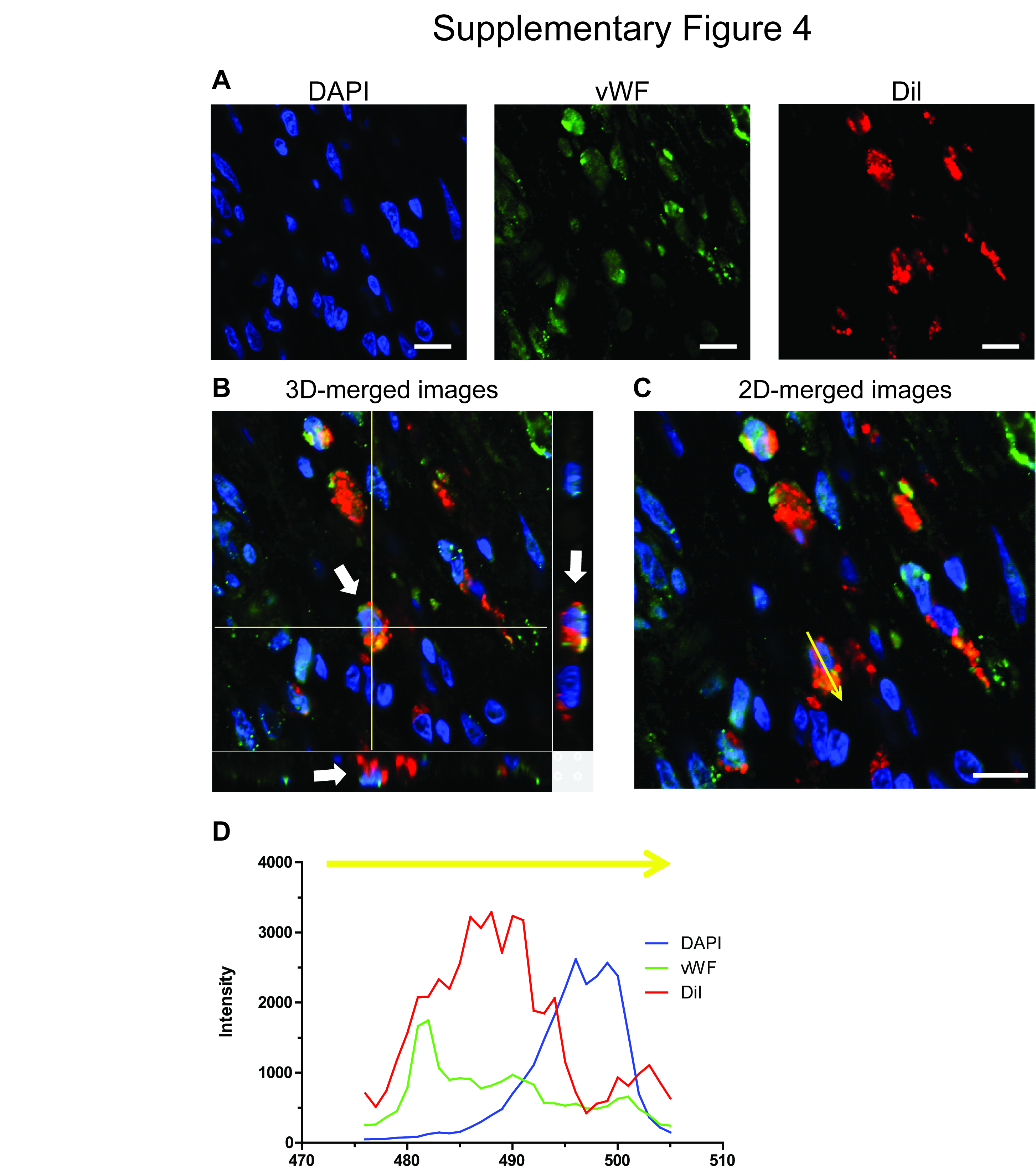

Supplement: S4 Fig — (A) Confocal laser scanning microscopy showing images of DAPI, vWF and DiI in separate or (B) 3D- or (C) 2D-merged. (D) Representative spectral overlap of DAPI, vWF and DiI in a cell indicated by an arrow in (C). Scale bar = 10 μm. (TIF) [file pone.0115430.s004.tif]
